# Quality Control of Natural Product Medicine and Nutrient Supplements

**DOI:** 10.1155/2013/182573

**Published:** 2013-07-16

**Authors:** Shuang-Qing Zhang, Feng Wei, Yu-Ming Fan, Feng Sun, Ying-Yong Zhao, Shuhua Bai

**Affiliations:** ^1^Department of Nutrition and Metabolism, National Institute for Nutrition and Food Safety, China Center for Disease Control and Prevention, Beijing 100050, China; ^2^Department of Traditional Chinese Medicine and Ethnic Medicine, National Institutes for Food and Drug Control, Beijing 100050, China; ^3^National Institutes for Food and Drug Control, Beijing 100050, China; ^4^Department of Obstetrics & Gynaecology, Yong Loo Lin School of Medicine, National University of Singapore, MD Block 11, CRC 03-09, 10 Medical Drive, Singapore 117597; ^5^Key Laboratory of Resource Biology and Biotechnology in Western China of Ministry of Education, College of Life Sciences, Northwest University, No. 229 Taibai North Road, Xi'an, Shaanxi 710069, China; ^6^Department of Basic Pharmaceutical Sciences, School of Pharmacy, Husson University, Bangor, ME 04401, USA

Natural product medicine (NPM) has been used to prevent and treat various human diseases in China, India, Japan, and other countries for centuries. Due to its long historical clinical uses and excellent therapeutic efficacy, NPM more and more attracts global attention, and many research institutes and pharmaceutical companies have been actively exploring NPM as a source for new drug discovery and development [1]. However, the characteristics of NPM are roles of multicomponents and multitargets due to its multiple components. If only few constituents are emphasized, the holistic characteristics of NPM will be ignored [2].

With the development of globalization and modernization of NPM, an important issue is the consistency and control ability of quality of NPM. Traditionally, identification of NPM is performed according to its morphological character, one or a few markers' thin-layer chromatography identification, and/or content determination [3, 4]. However, these methods do not provide a complete profile of the NPM, so it can't distinguish NPM with similar appearance and/or similar main chemical constitutions.

The characteristics of action of NPM are synergistic action of multicomponents and multitargets [5]. By a combination of multiple components, NPM may produce its beneficial effects by interacting with different cell signaling pathways and biological networks, achieving the same therapeutic efficacy of a normal isolated compound at much lower doses of isolated compounds [6]. Thus, a comprehensive method which could reflect the variation of most constituents in the NPM is necessary, especially the variation correlating with pharmacological and clinical efficacy. With the development of various modern technologies, advanced chemical, pharmacological, and biological technologies have facilitated an increasing number of researchers in the search for possible ways to explore the potential healthcare benefits of this multicomponents interaction system. In recent years, the analysis of NPM has begun to emphasize more on the integrative and holistic properties of NPM [7]. The chromatographic or spectroscopic fingerprint and multicomponents quantification have been used for the quality control of NPM in the past ten years. Based on some chromatographic or spectroscopic methods, the properties of absorption, distribution, metabolism, and excretion of NPM have been analyzed to screen the bioactive markers. In addition, the genomics and proteomics have been applied to the investigation of NPM. The different analytical techniques and methods of NPM are shown in Table 1.

In this special issue of quality control of NPM and nutrient supplements, 15 research papers have been published by the previously mentioned chromatographic or spectroscopic methods and contributed to quality control of NPM. These papers focused on the quantification analysis of multiple chemical components, bioactivity and their mechanism of actions of chemical components, and pharmacokinetics and tissue distribution studies.

Firstly, multicomponents determinations play a critical role in evaluating the quality of NPM and understanding the synergistic action of multicomponents and multitargets. Multicomponents quantitative methods of NPM were reported in this special issue. Among the analytical methods for NPM, HPLC with easy operation, high accuracy, and wide suitability is still the most popular method for the qualitative and quantitative analyses of NPM. For example, simultaneous quantification of limonin and six alkaloids in *Evodia rutaecarpa* has been completed by HPLC-DAD method. The results indicated that the quality control of *Evodia rutaecarpa* could be simplified to the measurement of four constituents and that limonin, 1-methyl-2-undecyl-4(1H)-quinolone, and dihydroevocarpine should also be served as the chemical markers together with evodiamine for the quality control of *Evodia rutaecarpa*.

Secondly, active components of NPM should be clarified by different methods in order to achieve better quality control of NPM and nutrient supplements. The bioactivities of pure compounds or crude extract form NPM have been evaluated by *in vitro* or *in vivo* experiments in this special issue. 

Last but not least, with the development of pharmacy, more and more researchers focus their attention on pharmacokinetics in drug discovery. Pharmacokinetics including absorption, distribution, metabolism, and excretion is regarded as the foundation of new drug discovery. Pharmacokinetics encompasses a broad spectrum of experiment and connotation. Five articles devoted to pharmacokinetics and identification of metabolites from pure compounds or crude extract of NPM. The complexity of single herbs or compound preparations causes diverse pharmacokinetics, and these articles provide key information for researchers in the future studies.

All in all, the published research papers will contribute to the development, improvement, validation, and/or extension of application of analytical methodology in the natural medicine sciences and nutrition supplements. Quality control of NPM has to establish reasonable analytical methods for analyzing the active constituents in NPM in order to clarify their therapeutic basis and mechanism of action more clearly. 

*Shuang-Qing Zhang* *Shuang-Qing Zhang* *Feng Wei* *Feng Wei* *Yu-Ming Fan* *Yu-Ming Fan* *Feng Sun* *Feng Sun* *Ying-Yong Zhao* *Ying-Yong Zhao* *Shuhua Bai* *Shuhua Bai*

## Figures and Tables

**Table 1:** The analytical techniques and methods of natural product medicine.

Analytical techniques	Chromatographic or spectroscopic methods
Thin-layer chromatography (TLC) techniques	High-performance TLC (HPTLC), microemulsion TLC (ME-TLC), and so forth
Gas chromatography (GC) techniques	GC-MS, two-dimensional GC (GC × GC), and so forth
High performance liquid chromatography (HPLC) techniques	HPLC-UV, HPLC-DAD, HPLC-ELSD, HPLC-FLD, HPLC-RID, HPLC-MS, HPLC-CEAD, HPLC-ESI/TOF-MS, UPLC-ESI/TOF-MS, and so forth
Capillary electrophoresis (CE) techniques	CE, capillary zone electrophoresis (CZE), micellar electrokinetic chromatography (MEKC), nonaqueous CE (NACE), capillary electrochromatography (CEC), and so forth
Infrared spectroscopy (IR) techniques	FT-IR, two-dimensional infrared (2D-IR), and so forth
Near-infrared spectroscopy (NIR) techniques	NIR, two-dimensional near-infrared (2D-NIR), and so forth
Nuclear magnetic resonance (NMR) techniques	Quantitative NMR (qNMR), diffusion-ordered spectroscopy (DOS) NMR, three-dimensional DOSY NMR, and so forth
